# A Systematic Review and Meta-Analysis of Autoantibodies for Diagnosis and Prognosis in Patients With Chronic Inflammatory Demyelinating Polyradiculoneuropathy

**DOI:** 10.3389/fnins.2021.637336

**Published:** 2021-05-24

**Authors:** Xiaoqian Guo, Lisha Tang, Qianyi Huang, Xiangqi Tang

**Affiliations:** Department of Neurology, The Second Xiangya Hospital, Central South University, Changsha, China

**Keywords:** CIDP, diagnosis, autoantibody, NF155, CNTN1

## Abstract

**Objectives:** To review the available evidence on sensitivity and specificity of anti-NF155 antibody detection in diagnosing a specific subset of patients with chronic inflammatory demyelinating polyradiculoneuropathy (CIDP) and to calculate the frequencies of different autoantibodies to paranodal proteins.

**Background:** Diagnosis of CIDP relies on clinical and neurophysiologic criteria and lacks useful diagnostic biomarkers. A subset of CIDP patients exhibit atypical clinical phenotypes and impaired response to conventional treatments. These patients were reported as having autoantibodies targeting paranodal protein neurofascin isoform 155 (NF155), contactin-1 (CNTN1), and contactin-associated protein-1 (CASPR1). Here, we conducted a meta-analysis to summarize evidence on the diagnostic and prognostic value of these autoantibodies, especially for anti-NF155 antibody.

**Methods:** We searched the following electronic bibliographic databases: PubMed, EMBASE, Cochrane Central Register of Controlled Trials (CENTRAL), and Web of Science. Eligible studies provided information to calculate the frequencies of anti-NF155 antibody and anti-CNTN1 antibody, the sensitivity and specificity of anti-NF155 antibody, and the incidence of improvement and deterioration among anti-NF155 antibody seropositive CIDP patients. Heterogeneity was assessed using Q and *I*^2^ statistics.

**Results:** The pooled frequency of anti-NF155 autoantibody across 14 studies was 7% [95% confidence interval (CI): 0.05–0.10] with high heterogeneity; the overall pooled sensitivity and specificity of anti-NF155 antibody for the diagnosis of a specific subgroup of CIDP patients were 0.45 (95% CI: 0.29–0.63) and 0.93 (95% CI: 0.86–0.97), respectively.

**Conclusions:** For diagnosing of a specific subset of CIDP characterized by poor response to intravenous immunoglobulin (IVIg), we found a moderate sensitivity and a high specificity. The anti-NF155 antibody test should be used as a confirmatory test rather than a screening test.

**Systematic Review Registration:** PROSPERO, identifier: CRD42020203385 and CRD42020190789.

## Introduction

Chronic inflammatory demyelinating polyradiculoneuropathy (CIDP) is a progressive paralyzing illness. The etiology of CIDP is unknown. The pathology of CIDP is complex. Based on the electrodiagnostic and pathological findings, the neuropathies of CIDP are conventionally classified as demyelinating neuropathy and axonal neuropathy (Latov, 2014).

The distinction between CIDP and other peripheral neuropathies may be challenging, considering the clinical and electrophysiological presentation may be quite heterogeneous.

The “nodopathy” was a novel concept introduced in recent studies. “Nodopathy” means the microstructural changes restricted to the nodal and paranodal regions induce significant nerve dysfunction (Kuwabara et al., 2017). Increasing evidence shows that nodopathy is associated with a subset of CIDP patients (Bunschoten et al., 2019).

**Figure 1:**
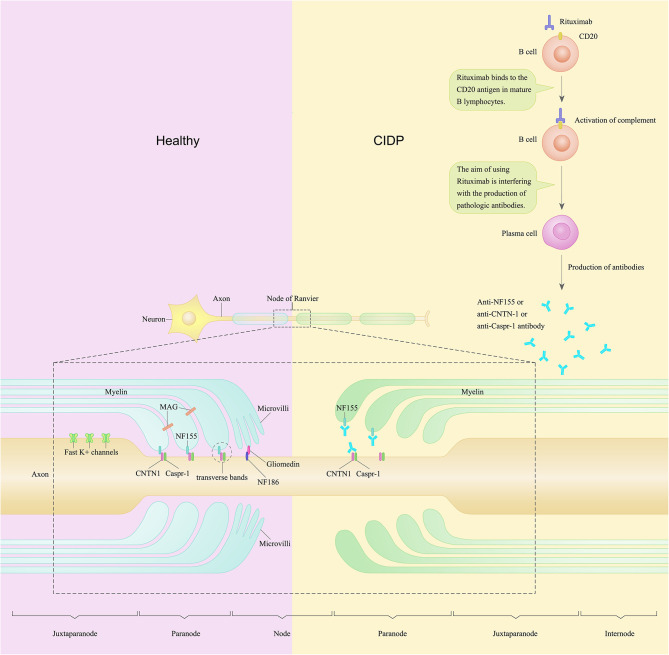
A schematic of the node of Ranvier and paranode in the peripheral nerve of a patient with chronic inflammatory demyelinating polyradiculoneuropathy compared to healthy control. In the peripheral nervous system, Schwann cells contact and wrap around axons and create polarized domains including the node, paranode, juxtaparanode, and internode. Neurofascin-155 (NF155) along with contactin-1 (CNTN1) and contactin-associated protein (CASPR1) form the complex named transverse bands. Transverse bands anchor loops of myelin to the axon at the paranode. In CIDP patients, anti-NF155 autoantibodies may bind to NF155 and disable NF155, thus cause a selective loss of the transverse bands at the paranode loops. Rituximab is a monoclonal antibody against CD20 and has been found to be efficacious in several cases of CIDP. The purpose of using rituximab is to interfere with the production of pathological autoantibodies in CIDP patients.

Recently, the disruption of axoglial junctions in the node/paranode has been observed in a subgroup of CIDP patients with antibodies against paranodal proteins, especially for neurofascin 155 (NF155) (Tang et al., 2020). In the lesioned paranodal region, detachment of the myelin loops may be caused by the absence of cell adhesion molecules, including NF155, contactin-1 (CNTN1), neurofascin 186 (NF186), and contactin-associated protein 1 (CASPR1). The detachment of the myelin loops may lead to secondary axonal degeneration (Kuwabara et al., 2017). The glial protein NF155 is associated with the axonal proteins CNTN1 and CASPR1. NF 155, CNTN 1, CASPR1 together form an axoglial complex in the paranodal area (Figure 1). NF186 is expressed at the nodal axolemma. NF186 interacts with gliomedin and the neuron-glia-related cell-adhesion molecule (NrCAM) (Manso et al., 2019; Tang et al., 2020). These nodal/paranodal proteins are important for the adhesion of myelin sheath borders to axons. A proportion of CIDP patients have antibodies against CNTN1 and NF155. In the peripheral nervous system (PNS) of this subgroup of CIDP patients, the function as adhesion receptors are severely disrupted (Wolbert et al., 2020).

This subgroup of patients show tremor, ataxia, and poor response to intravenous immunoglobulin (IVIg) as a distinct clinical presentation. These patients also show conduction blocks and decrease of compound muscle action potentials in the electrophysiological studies (Pascual-Goñi et al., 2019). Moreover, some seropositive CIDP patients show remarkable improvement after treatment with rituximab (Bunschoten et al., 2019). One hypothesis of the pathogenic mechanisms of this subset of CIDP patients is that, in this subgroup of CIDP patients, anti-NF155 antibodies may bind to NF155 and disable NF155, thus cause a selective loss of the transverse bands at the paranode loops. Rituximab, works as a monoclonal antibody against CD20, may interfere with the production of anti-NF155 autoantibodies in these CIDP patients (Figure 1).

Treatment response among these CIDP patients should be mentioned regarding the therapeutic guiding value of these autoantibodies. Treatment responses in CIDP are usually evaluated by neurological or electrophysiological examinations. It turns out from several observational studies (Kawamura et al., 2013; Doppler et al., 2016; Burnor et al., 2018; Zhang et al., 2019; Godil et al., 2020; Muley et al., 2020) that the treatment of this subset of CIDP patients need to be individualized.

Over the last 5 years, a series of studies have reiterated the clinical importance of distinguishing the subset of patients with CIDP with autoantibodies against the nodal/paranodal proteins, further emphasizing the need for optimal therapeutic decisions to prevent secondary axonal degeneration led by axoglial disjunction (Querol et al., 2017; Bunschoten et al., 2019; Zhang et al., 2019; Cortese et al., 2020).

The diagnosis of CIDP is based on clinical features, nerve conduction studies, MRI, nerve biopsy, spinal fluid analysis, and nerve ultrasound studies. Because there is no specific definitive biomarker to diagnose CIDP, misdiagnosis is frequent (Ogata et al., 2015; Van Den Bergh et al., 2020). Moreover, the disease needs to be recognized as early as possible, and effective management needs to be arranged early to prevent secondary axonal degeneration and minimize disability from axonal degeneration.

Some studies support NF155 antibodies may identify a CIDP phenotype characterized by severe polyradiculoneuropathy, poor response to IVIg, and disabling tremor associated with NF186, CNTN1, and CASPR1, respectively. However, the evidence for NF186 is not as strong when compared to NF155. To date, there is limited evidence to recommend the systematic use of autoantibodies as potential diagnostic biomarkers. Larger multicenter retrospective observational studies and systematic reviews are required for two reasons: (1) early diagnosis of this subgroup of CIDP; (2) identification of biomarkers that predict responsiveness.

Definitive evidence is required to provide quantitative data on gaps in knowledge and to inform future research efforts. Here, we focus on integrating the published evidence systematically to evaluate antibodies against paranodal proteins as potential diagnostic and prognostic biomarkers for CIDP.

The objectives of this systematic review and meta-analysis are as follows: To review the available evidence on the sensitivity and specificity of anti-NF155 antibody in diagnosing CIDP; to review the available evidence on sensitivity and specificity of NF155 in diagnosing a specific subset of CIDP patients; to calculate the frequencies of NF155 and CNTN1, and to explore the association between anti-NF155 antibody and the prognosis of patients with CIDP. The study are limited due to a small sample size. Thus, we only focus on NF155 and CNTN1 at this time. We give up analyzing the values of CASPR1 and NF186 due to underreporting.

## Methods

The protocol of this systematic review was registered with a prospective international registry of systematic reviews (PROSPERO) (CRD42020203385 and CRD42020190789) and formulated according to a statement for preferred reporting items for systematic review and meta-analysis protocols (PRISMA-P). Protocol CRD42020203385 documents the review method of the diagnostic section of this systematic review and meta-analysis and acts as a safeguard against arbitrary decision-making during review implementation. Protocol CRD42020190789 constitutes the preliminary planning and methodical documentation of the prognostic section for this systematic review and meta-analysis.

### Literature Search Strategy

This review is reported in accordance with the Preferred Reporting Items for Systematic Reviews and Meta-analyses (PRISMA) guidelines. We searched the following electronic bibliographic databases: PubMed, EMBASE, Cochrane Central Register of Controlled Trials (CENTRAL), and Web of Science for all published work from January 1st, 1974 to August 15th, 2020. The search string was as follows: (chronic inflammatory demyelinating polyneuropathy OR CIDP) AND (autoantibody OR neurofascin 155 OR contactin-1OR neurofascin 186 OR CASPR1). The studies were restricted to those conducted on humans and reported in English. Reference lists and articles citing the relevant publications were reviewed for all relevant articles after the full-text screening. Citation alerts were set up using the Web of Science service.

### Inclusion and Exclusion Criteria

The diagnosis of participants from the included studies of our systematic review met the European Federation of Neurological Societies/Peripheral Nerve Society (EFNS/PNS) criteria.

We included cohort studies, case-control studies, cross-sectional studies, and case series to conduct a single-arm meta-analysis to calculate the frequencies of anti-NF155 antibody and anti-CNTN1 antibody.

For the diagnostic portion of this review, we included cohort studies and cross-sectional studies to measure the accuracy of anti-NF155 antibody test for the diagnosis of a specific subset of CIDP characterized by poor response to IVIg. These studies contain data to generate a two-by-two table listing true positive (TP), true negative (TN), false positive (FP), and false negative (FN) rates.

For the prognostic portion of this review, we included cohort studies, case-control studies, and case series to obtain sufficient follow-up data. Our main focus was CIDP patients with anti-NF155 antibody in this portion.

### Data Extraction

XG and LT extracted the following data into a specially designed form: (1) author, year of publication, and journal; (2) study design; (3) study population and participants; (4) reference standard used: EFNS/PNS criteria to diagnose CIDP; (5) IVIg treatment response; (6) methodological description of ELISA, cell-based binding assay, western blot analysis, and teased nerve fiber binding assay; (7) QUADAS items (see below); (8) data on diagnosis (reference standard results) and results for autoantibodies for the two-by-two table; (9) number of patients meeting the EFNS/PNS criteria.

### Quality Assessment

XG and LT independently assessed the risk of bias in the included studies by utilizing the Quality Assessment of Diagnostic Accuracy Studies-2 (QUADAS-2) tool (Whiting et al., 2011). This assessment tool contains 4 domains and 14 questions and is the most advanced tool to evaluate risk bias from diagnosis validity. Assessment results were illustrated in both figures and tables.

### Statistical Analyses

All analyses were performed by RevMan 5.4.1 (The Cochrane Collaboration, Oxford, UK) and STATA/MP 16.0 (StataCorp, College Station, TX).

#### Statistical Analysis and Data Synthesis

We conduct a single-arm meta-analysis to calculate the frequencies of anti-NF155 antibody and anti-CNTN1 antibody. A single-arm meta-analysis is applicable to calculate the pooled effect of incidence rates of events as well as the pooled prevalence. Random effects model was used to cover the variation between and within included studies.

For the diagnosis section, two-by-two tables were generated to calculate the sensitivity and specificity. We presented individual study results graphically by plotting estimates of sensitivities and specificities as a forest plot. We meta-analyzed pairs of sensitivity and specificity using a bivariate random-effects model. This method estimates a summary sensitivity and specificity of the test while taking into account the correlation between sensitivity and specificity.

For the prognosis section, we listed all common prognosis outcomes, such as improvement, recovery, recurrence, deterioration, complication, disability, and death. We conducted a single-arm meta-analysis to obtain the incidence rate of each outcome.

#### Investigations of Heterogeneity

Heterogeneity was assessed by using Q and *I*^2^ statistics. We considered an *I*^2^ > 50% indicative of substantial heterogeneity.

## Results

### Literature Selection

**Figure 2:**
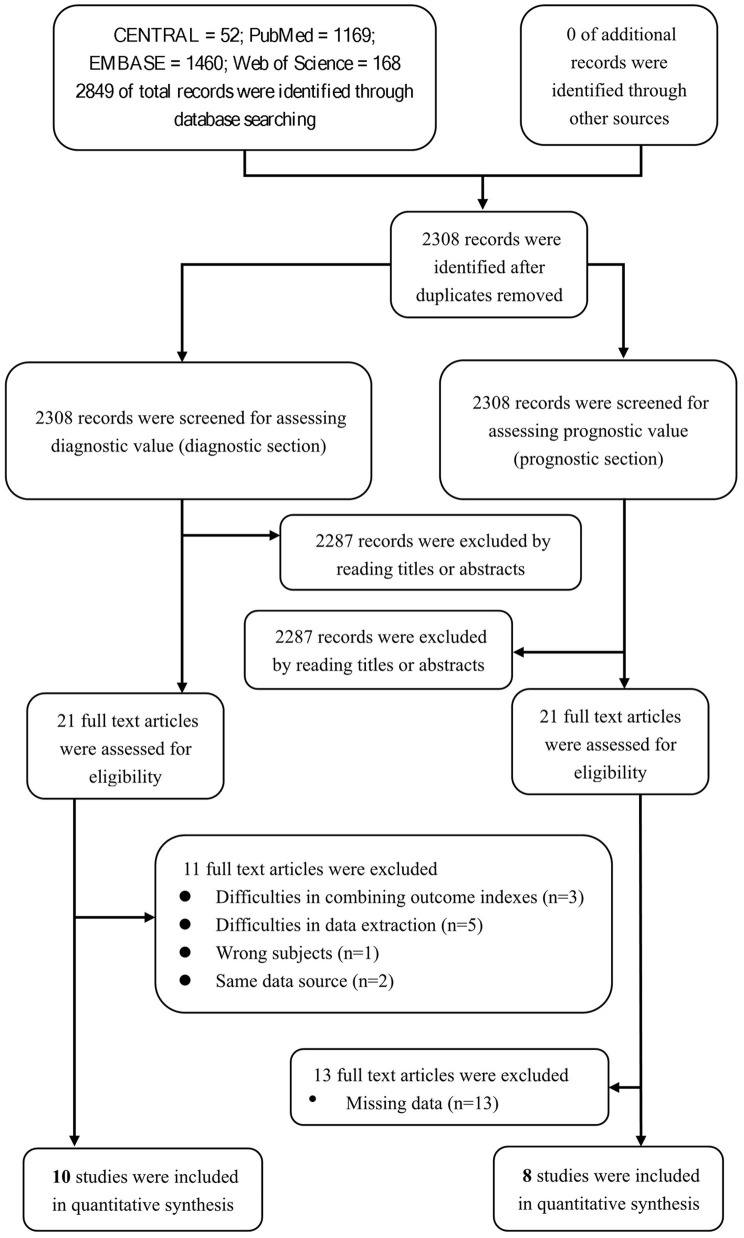
Study flow diagram.

We aimed to review the available evidence on the sensitivity and specificity of the nodal/paranodal autoantibodies in diagnosing CIDP. The database searches yielded 2,849 entries, of which 541 were excluded because of duplications, reviews, or irrelevance. Of the remaining 2,308 studies, 2,287 studies were excluded through screening the titles or abstracts. Eleven studies of the 21 remaining studies were excluded after reading the full texts for the diagnostic section. Among these 11 studies, 3 were excluded for difficulties in combining outcome indexes (Querol et al., 2013; Doppler et al., 2015, 2016), 5 were excluded for difficulties in data extraction (Ogata et al., 2019; Zhang et al., 2019; Godil et al., 2020; Kouton et al., 2020; Muley et al., 2020), 1 was excluded for wrong subjects (Vallat et al., 2020), and 2 were excluded for same data source (Miura et al., 2015; Delmont et al., 2017). We enrolled 10 studies for a qualitative systematic review in the diagnostic section to assess the value of neurofascin antibodies in diagnosing CIDP. Among these 10 studies, 4 were included for the overall pooled sensitivity and specificity for diagnosing the subset of CIDP patients with poor response to IVIg using anti-NF155 autoantibody (Figure 2).

Twenty-one studies were assessed for the prognostic section of our systematic review. Among these 21 studies, 13 studies (Kawamura et al., 2013; Yan et al., 2014; Doppler et al., 2015, 2016; Miura et al., 2015; Ogata et al., 2015; Devaux et al., 2016; Delmont et al., 2017; Mathey et al., 2017; Zhang et al., 2019; Cortese et al., 2020; Kouton et al., 2020; Vallat et al., 2020) were excluded due to missing data, and 8 studies were enrolled due to their quantitatively integrated relationship between prognosis and anti-NF155 antibody (Figure 2).

Twenty studies of the 21 full-text downloaded studies were identified for calculating the frequencies of different autoantibodies to paranodal proteins including a total of 3,605 patients (**Table 5**).

Ongoing trials should be conducted in the future to update this integrated evidence. We excluded a 2019 paper regarding negative test results of nodal/paranodal autoantibodies (Vallat et al., 2020). That paper contains information about autoantibodies against myelin areas other than the node/paranode area.

### Frequencies of Autoantibodies

**Figure 3:**
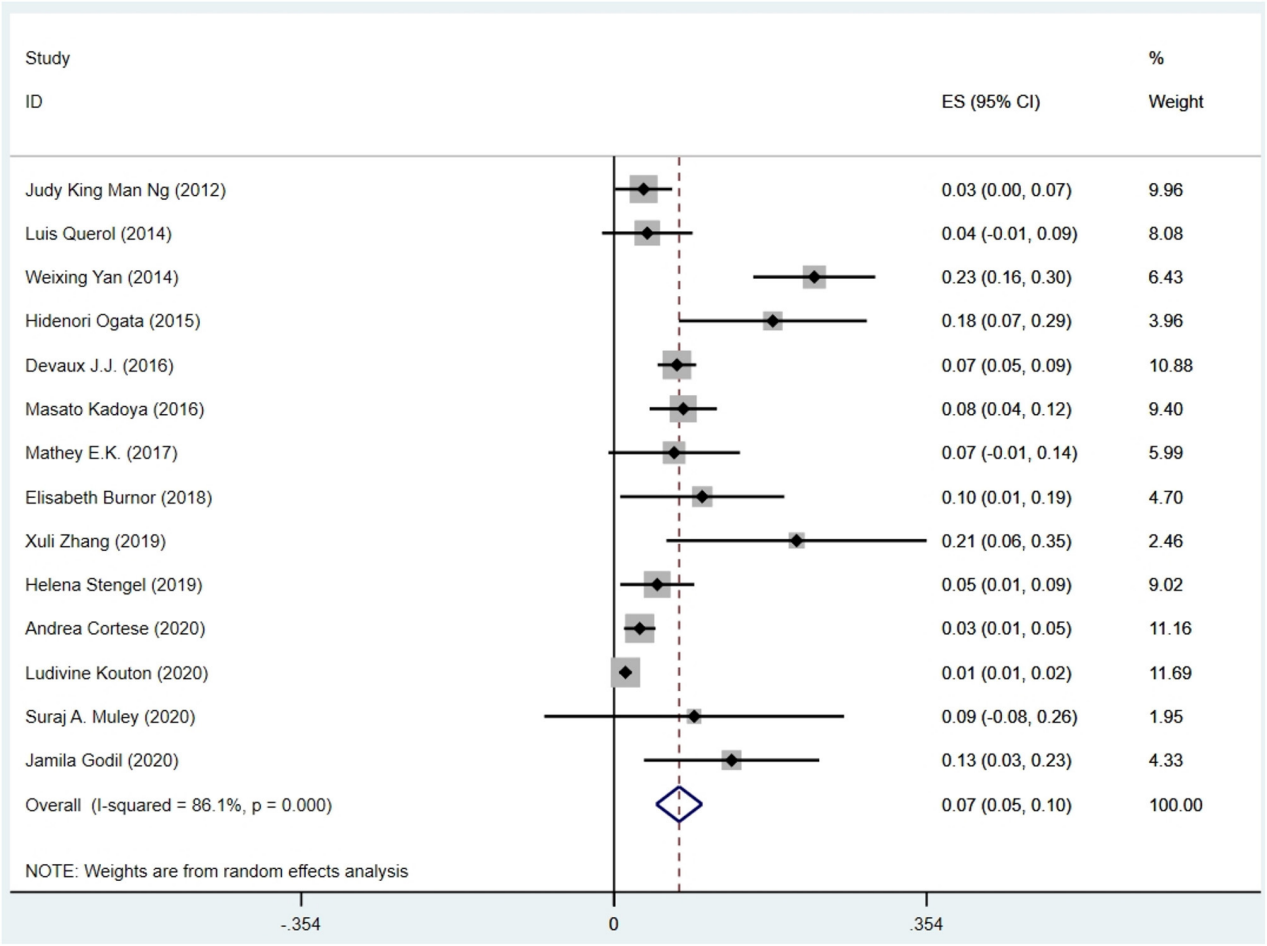
The forest plot of the frequency of anti-NF155 autoantibody across 14 studies.

The pooled effect size was 7% [95% confidence interval (CI) 0.05–0.10], which here refers to the frequency of anti-NF155 autoantibody across 14 studies (Ng et al., 2012; Querol et al., 2014; Yan et al., 2014; Ogata et al., 2015; Devaux et al., 2016; Kadoya et al., 2016; Mathey et al., 2017; Burnor et al., 2018; Stengel et al., 2019; Zhang et al., 2019; Cortese et al., 2020; Godil et al., 2020; Kouton et al., 2020; Muley et al., 2020). The *p*-value for the z statistic was 0.000 (<0.05), which reflects statistical significance. The pooled outcome measures were determined using random-effects models described by DerSimonian and Laired because of high heterogeneity (*I*^2^ = 86.1% >50%). Based on this high heterogeneity, we are uncertain about the conclusion that the proportion of patients with anti-NF155 autoantibody is 7% (Tables 1, 2 and Figure 3).

**Table 1:** Percentage of CIDP patients with anti-NF155 autoantibodies.

**References**	**Number of NF155 positive CIDP patients**	**Number of CIDP patients**	**Antibody positive rate**
Ng et al. (2012)	4	119	3%
Querol et al. (2014)	2	53	4%
Yan et al. (2014)	32	141	23%
Ogata et al. (2015)	9	50	18%
Devaux et al. (2016)	38	533	7%
Kadoya et al. (2016)	15	191	8%
Mathey et al. (2017)	3	44	7%
Burnor et al. (2018)	4	40	10%
Zhang et al. (2019)	6	29	21%
Stengel et al. (2019)	5	102	5%
Cortese et al. (2020)	10	342	3%
Kouton et al. (2020)	13	1,000	1%
Muley et al. (2020)	1	11	9%
Godil et al. (2020)	6	45	13%

**Table 2:** The illustration of Figure 3.

**References**	**ES (effect size)**	**[95% Conf. Interval]**		**% Weight**
Ng et al. (2012)	0.034	0.001	0.066	9.96
Querol et al. (2014)	0.038	−0.014	0.089	8.08
Yan et al. (2014)	0.227	0.158	0.296	6.43
Ogata et al. (2015)	0.180	0.074	0.286	3.96
Devaux et al. (2016)	0.071	0.049	0.093	10.88
Kadoya et al. (2016)	0.079	0.040	0.117	9.40
Mathey et al. (2017)	0.068	−0.006	0.143	5.99
Burnor et al. (2018)	0.100	0.007	0.193	4.70
Zhang et al. (2019)	0.207	0.059	0.354	2.46
Stengel et al. (2019)	0.049	0.007	0.091	9.02
Cortese et al. (2020)	0.029	0.011	0.047	11.16
Kouton et al. (2020)	0.013	0.006	0.020	11.69

*Heterogeneity chi-squared = 93.65 (df = 13); p = 0.000; I-squared (variation in ES attributable to heterogeneity) = 86.1%; Estimate of between-study variance Tau-squared = 0.0015; Test of ES = 0: z = 5.57, p = 0.000*.

**Figure 4:**
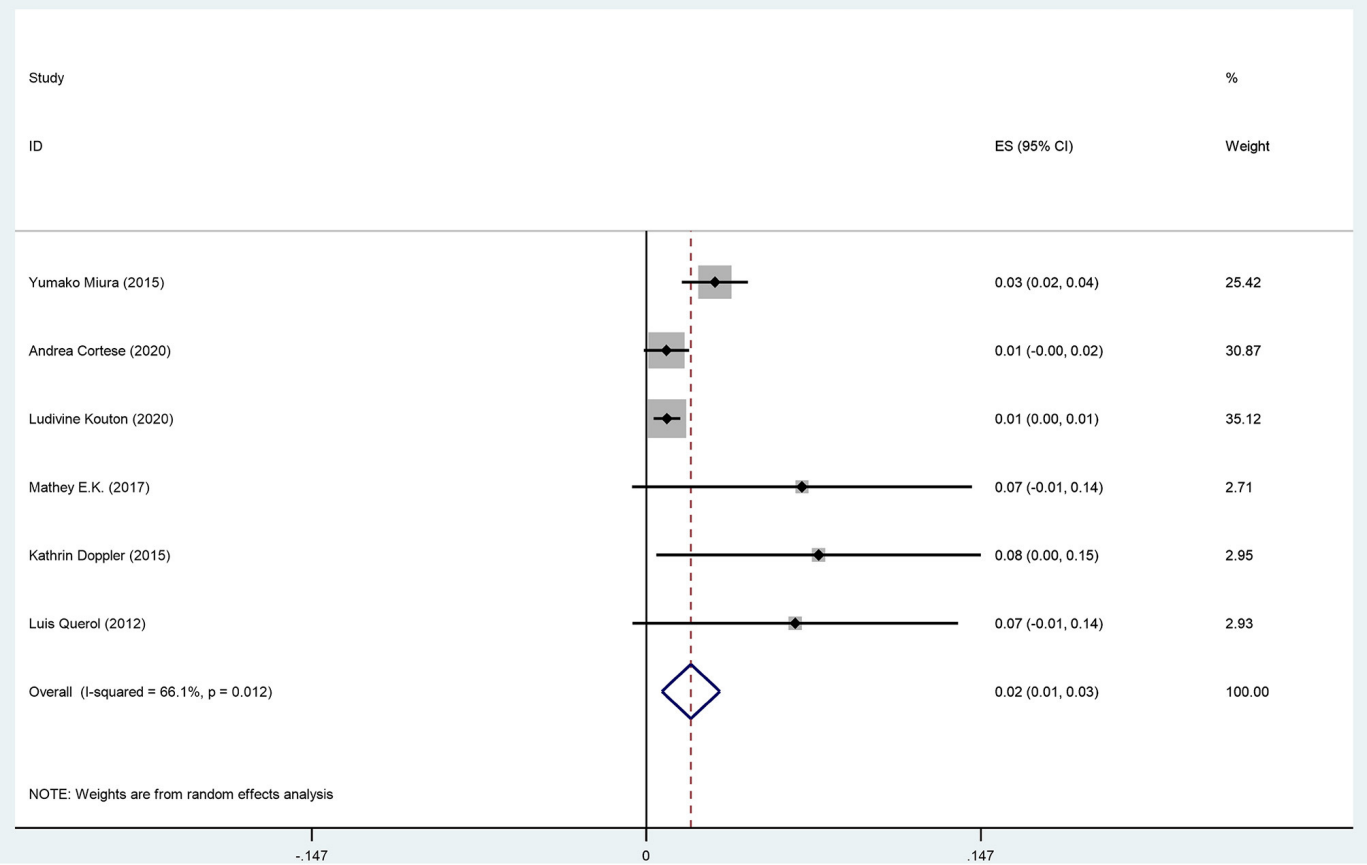
The forest plot of the frequency of anti-CNTN1 autoantibody across 6 studies.

The pooled effect size is 2% (95% CI: 0.01–0.03), which refers to the frequency of anti-CNTN1 autoantibodies across 6 studies (Querol et al., 2013; Doppler et al., 2015; Miura et al., 2015; Mathey et al., 2017; Cortese et al., 2020; Kouton et al., 2020). The *p*-value for the z statistic was 0.000 (<0.05), which reflects statistical significance. The pooled outcome measures were determined using random-effects models as described by DerSimonian and Laired because of high heterogeneity (*I*^2^ = 66.1% >50%). Based on this high heterogeneity, we are uncertain about the conclusion that the proportion of patients with anti-CNTN1 is 2% (Tables 3, 4 and Figure 4).

**Table 3:** Percentage of CIDP patients with anti-CNTN1 autoantibodies.

**References**	**Number of CNTN1 positive CIDP patients**	**Number of CIDP patients**	**Antibody positive rate**
Querol et al. (2013)	3	46	7%
Miura et al. (2015)	16	533	3%
Doppler et al. (2015)	4	53	8%
Mathey et al. (2017)	3	44	7%
Cortese et al. (2020)	3	342	1%
Kouton et al. (2020)	9	1,000	1%

**Table 4:** The illustration of Figure 4.

**References**	**ES (effect size)**	**[95% Conf. Interval]**		**% Weight**
Querol et al. (2013)	0.065	−0.006	0.137	2.93
Miura et al. (2015)	0.030	0.016	0.045	25.42
Doppler et al. (2015)	0.075	0.004	0.147	2.95
Mathey et al. (2017)	0.068	−0.006	0.143	2.71
Cortese et al. (2020)	0.009	−0.001	0.019	30.87
Kouton et al. (2020)	0.009	0.003	0.015	35.12
D+L pooled ES	0.019	0.007	0.032	100.00

*Heterogeneity chi-squared = 14.74 (df = 5); p = 0.012; I-squared (variation in ES attributable to heterogeneity) = 66.1%; Estimate of between-study variance Tau-squared = 0.0001; Test of ES = 0: z = 3.00, p = 0.003*.

### Study Characteristics

The main characteristics of the eligible studies for the following review questions are listed in Table 5: What are the frequencies of autoantibodies to paranodal proteins? What is the association between these autoantibodies and the diagnosis of patients with CIDP? What is the association between these autoantibodies and the prognosis of CIDP patients? There are a total of 20 studies listed in Table 5 (Ng et al., 2012; Kawamura et al., 2013; Querol et al., 2013, 2014; Yan et al., 2014; Doppler et al., 2015, 2016; Miura et al., 2015; Ogata et al., 2015, 2019; Devaux et al., 2016; Kadoya et al., 2016; Delmont et al., 2017; Mathey et al., 2017; Burnor et al., 2018; Zhang et al., 2019; Cortese et al., 2020; Godil et al., 2020; Kouton et al., 2020; Muley et al., 2020).

**Table 5:** Study characteristics.

**References**	**Countries**	**Type of antibody**	**Partici-pants with CIDP**	**Antibody positive patients**	**Male (female)**	**Age at onset mean (range)**	**Sample source**	**Method for testing autoantibody**	**Treatment (*n*)**
Ng et al. (2012)	Japan, Sweden, Germany	NF155	119	4	Unknown	Unknown	Serum	ELISA; CBA	Corticosteroids; IVIg; Plasma exchange
Querol et al. (2013)	Spain	CNTN1 CASPR1	46	3 (CNTN1) 1 (CASPR1)	Among seropositive patients: 1 (2)	Among seropositive patients: 71; Among seronegative patients: 51.6	Serum	CBA; Western blot analysis	Corticosteroids; IVIg; Plasma exchange
Kawamura et al. (2013)	Japan	NF155	23	10	Among CCDP patients: 3 (4)	Among CCDP patients: 28.9 (16-48)	Serum CSF	ELISA; CBA	Corticosteroids; IVIg; Plasma exchange; Azidothymidine; Interferon β-1a/b
Querol et al. (2014)	Spain	NF155	53	2	Among seropositive patients: 2 (0)	Among seropositive patients: 34; Among seronegative patients: Unknown	Serum	CBA; Teased nerve fiber binding assay; ELISA	Corticosteroids; IVIg; Plasma exchange
Yan et al. (2014)	Sydney, Australia, Japan, and China	NF155	141	32	Unknown	Unknown	Serum	ELISA;	Unknown
Ogata et al. (2015)	Japan	NF155	50	9	Unknown	Among seropositive patients: 25.2 (13–50); Among seronegative patients: 47.9 (13–76)	Serum	Flow cytometric assay; CBA Teased nerve fiber binding assay	Corticosteroids; IVIg; Plasma exchange
Doppler et al. (2015)	Germany	CNTN1	53	4	43 (10)	Unknown	Serum Plasma	ELISA; CBA	Unknown
Miura et al. (2015)	Japan	CNTN1	533	16	Unknown	Among seropositive patients: 60 (33–81); Among seronegative patients: 52 (22–66)	Serum	ELISA; CBA	Corticosteroids (22); IVIg (29); Plasma exchange
Kadoya et al. (2016)	Japan	NF155	191	15	Among seropositive patients: 11 (4)	Among seropositive patients: 32; Among seronegative patients: 50	Serum	ELISA; CBA	Corticosteroids (43); IVIg (58); Plasma exchange (11)
Doppler et al. (2016)	Germany	CASPR1	35	1	Among seropositive patients: 1 (0)	Among seropositive patients: 30; Among seronegative patients: Unknown	Serum	CBA; Teased nerve fiber binding assay	Corticosteroids; IVIg; Plasma exchange; Rituximab
Devaux et al. (2016)	Japan	NF155	533	38	Among sero-positive patients: 27 (11)	Among seropositive patients: 31 (10–67); Among seronegative patients: 48 (6–83)	Serum	ELISA; CBA; Teased nerve fiber binding assay	Corticosteroids; IVIg
Mathey et al. (2017)	Canada, Australia	NF155 CNTN1	44	3 (NF155) 3 (CNTN1)	Among sero-positive patients: 4 (2) Among sero-negative patients: 25 (13)	Among seropositive patients: 42 (NF155), 53 (CNTN1); Among seronegative patients: 61.4	Serum	ELISA; CBA; Teased nerve fiber binding assay	Unknown
Delmont et al. (2017)	France, Spain, Italy, and Singapore	NF140 NF186	246	5	Among seropositive patients: 3(2)	Among seropositive patients: 61 (2–70); Among seronegative patients: 58 (22–82)	Serum	CBA	Corticosteroids; IVIg; Plasma exchange
Burnor et al. (2018)	US	NF155	40	(NF155) 1 (NF186)	Among sero-positive patients: 3 (2)	Unknown	Serum	CBA	Corticosteroids; IVIg; Plasma exchange; Rituximab; Cyclophosphamide
Ogata et al. (2019)	Japan	NF155	71	35	50 (21)	Among seropositive patients: 25 (13–64) Among seronegative patients: 46 (10–76)	Serum	Flow cytometry	Corticosteroids (35); IVIg (21); Plasma exchange (5); Other immunotherapies (8)
Zhang et al. (2019)	China	NF155 NF186	29	6 (NF155) 1 (NF186)	17 (12)	Among seropositive patients: 38.1 (28–64); Among seronegative patients: 47.5 (16–70)	Serum	CBA; Teased nerve fiber binding assay	Corticosteroids (28); IVIg (11); Plasma exchange (3); Rituximab (1)
Cortese et al. (2020)	Italy	NF155 CNTN1 CASPR1	342	10 (NF155) 3 (CNTN1) 6 (CASPR1)	Among sero-positive patients: 11 (7)	Among seropositive patients: 36 (13–82) Among seronegative patients: 39 (18–64)	Serum	ELISA; CBA	Corticosteroids (51); IVIg (72); Plasma exchange (16)
Kouton et al. (2020)	France, Belgium, Switzerland	NF155 CNTN1	1,000	13 (NF155) 9 (CNTN1)	Among seropositive patients: 14 (8) Among seronegative patients: 26 (14)	Among NF155+ patients: 56 Among CNTN1+ patients: 63 Among seronegative patients: 62	Serum	CBA; Flow cytometry	Unknown
Muley et al. (2020)	US	NF155	11	1	3 (8)	Unknown	Serum	Unknown	Corticosteroids (11); IVIg (11); Plasma exchange (7); Mycophenolate mofetil (4); Azathioprine (2); Rituximab (11)
Godil et al. (2020)	US	NF155	45	6	36 (9)	Unknown	Serum	Western blot analysis	Corticosteroids (17); IVIg (37); Plasma exchange (8); Cyclophosphamide (7); Rituximab (6)

*NF155, neurofascin 155; NF186, neurofascin 186; CNTN1, contactin 1; CASPR1, contactin-associated protein 1; ELISA, enzyme-linked immunosorbent assays; CBA, cell-based assay; IVIg, intravenous immunoglobulin*.

Among these 20 studies, 5 studies were conducted in multiple countries, and the remaining 15 studies were single-country studies. Among the single-country studies, 6 were conducted in Japan, 3 were conducted in the United States, 2 were conducted in Spain, 2 were conducted in Germany, 1 was conducted in Italy, and 1 was conducted in China. The diversity of the results may be influenced by ethnicity.

In 5 included studies, seropositive patients who were refractory to conventional therapy received rituximab. Godil et al. found an NF155 Ig4 seropositive patient improved after taking rituximab (Godil et al., 2020). In the study of Burnor et al., 2 NF155 seropositive patients markedly improved and 1 NF155 seropositive patient slightly improved after receiving rituximab (Burnor et al., 2018). In the study of Muley et al., at least 1 seropositive patient responded to rituximab (Muley et al., 2020). In the report of Zhang et al., rituximab was effective on one seropositive patient (Zhang et al., 2019). Besides the studies included in Table 5, we have found 2 studies that report the efficacy of rituximab against anti-CNTN1 antibody-positive CIDP (Querol et al., 2015; Delmont et al., 2020). As shown in Querol's research (Querol et al., 2015), one anti-CNTN1-positive patient was severely disabled and improved dramatically after rituximab treatment and was able to be withdrawn from other treatments. As shown in Delmont's research (Delmont et al., 2020), 6 CIDP patients with IgG4 anti-CNTN1-antibodies were resistant to first-line CIDP treatments and received rituximab. Of these 6 patients, 5 obtained efficacy (83%). All of these indicate that CIDP patients with CNTN1 and NF155 antibodies which were not satisfied with IVIg treatment may potentially respond well to rituximab.

In addition, cyclophosphamide was mentioned in 2 included studies. Azidothymidine was reported in 2 included studies. Interferon β and mycophenolate mofetil were each reported in 1 study.

Table 5 also summarizes the clinical heterogeneity. Gender, sample source, and method for testing autoantibody may introduce bias and affect the validity of our conclusions.

### Diagnosis

#### QUADAS-2 Quality Assessment

**Figure 5:**
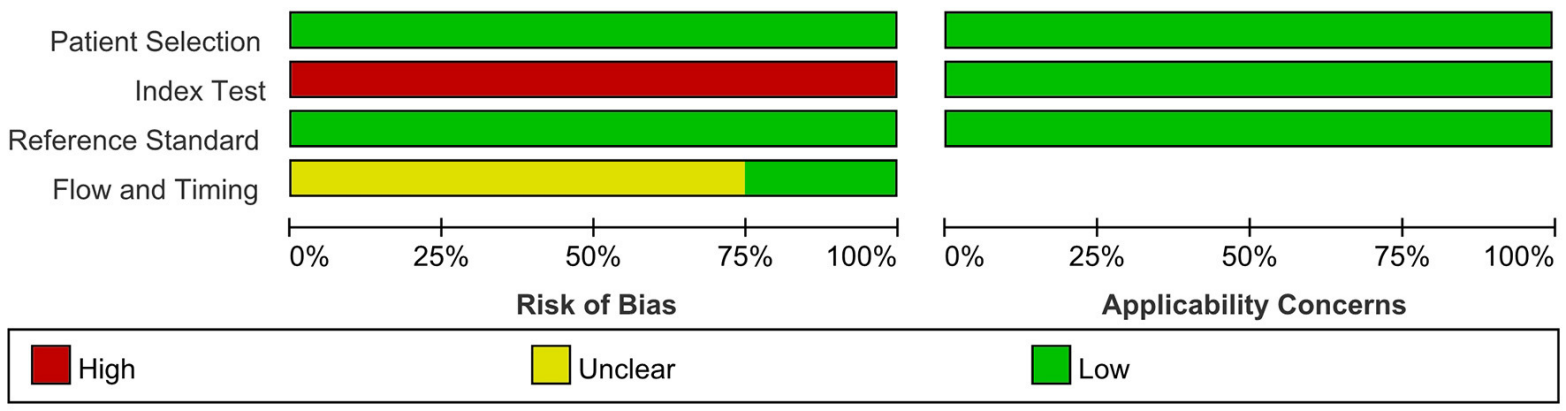
Risk of bias and applicability concerns graph: review authors' judgements about each domain presented as percentages across included studies.

We provide details of the risk of bias for 4 studies (Devaux et al., 2016; Kadoya et al., 2016; Zhang et al., 2019; Cortese et al., 2020) in Table 6 using QUADAS-2 quality assessment items. Figure 5 also presents a summary of the risk of bias for all included studies. The QUADAS-2 quality assessment tool comprises four domains: participant selection, index test, reference standard, and flow and timing. There is an overall low risk in two of the domain categories of QUADAS-2 (patient selection, reference standard), a high risk of bias for the domain index test, and an unclear risk for the flow and timing.

**Table 6:** QUADAS-2 criteria for included studies.

**References**	**Risk of bias**											**Concerns regarding applicability**		
	**Patient selection**			**Index test**		**Reference standard**		**Flow and timing**				**Patient selection**	**Index test**	**Reference standard**
	**Q1**	**Q2**	**Q3**	**Q1**	**Q2**	**Q1**	**Q2**	**Q1**	**Q2**	**Q3**	**Q4**			
Devaux et al. (2016)	Y	Y	Y	N	U	Y	Y	U	Y	Y	Y	N	N	N
Kadoya et al. (2016)	Y	Y	Y	N	U	Y	Y	U	Y	Y	Y	N	N	N
Zhang et al. (2019)	Y	Y	Y	N	U	Y	Y	U	Y	Y	Y	N	N	N
Cortese et al. (2020)	Y	Y	Y	N	U	Y	Y	U	Y	Y	Y	N	N	N

*QUADAS-2, Quality Assessment of Diagnostic Accuracy Studies-2*.

*Y, Yes; U, Unclear; N, No; Patient selection domain: Q1 = Was a consecutive or random sample of patients enrolled? Q2 = Was a case-control design avoided? Q3 = Did the study avoid inappropriate exclusions? Index test domain: Q1 = Were the index test results interpreted without knowledge of the results of the reference standard? Q2 = If a threshold was used, was it pre-specified? Reference standard domain: Q1 = Is the reference standard likely to correctly classify the target condition? Q2 = Were the reference standard results interpreted without knowledge of the results of the index test? Flow and timing domain: Q1 = Were there an appropriate interval between index test(s) and reference standard? Q2 = Did all patients receive a reference standard? Q3 = Did patients receive the same reference standard? Q4 = Were all patients included in the analysis?*

The main source of bias arose from the domain index test, which uses one of the following methods to determine the presence of autoantibodies in the samples of enrolled patients: ELISA; cell-based binding assay; western blot analysis; flow cytometry. The reference standard is used to diagnose CIDP via the criteria of EFNS/PNS by a clinician. Patients enrolled in all included retrospective studies knew the reference standard results before taking the index test, which carried a high risk of bias.

For the reference standard domain described above, the results were not influenced by the index test results. Moreover, the overall risk of domain flow and timing is unclear because the appropriate interval between the index test and reference standard is uncertain from the perspective of us, the two authors who extracted the data.

The result of the QUADAS-2 assessment refers only to the quality of evidence on the sensitivity and specificity of anti-NF155 autoantibodies in diagnosing a subset of CIDP.

#### Diagnosing a Specific Subgroup of CIDP

**Figure 6:**
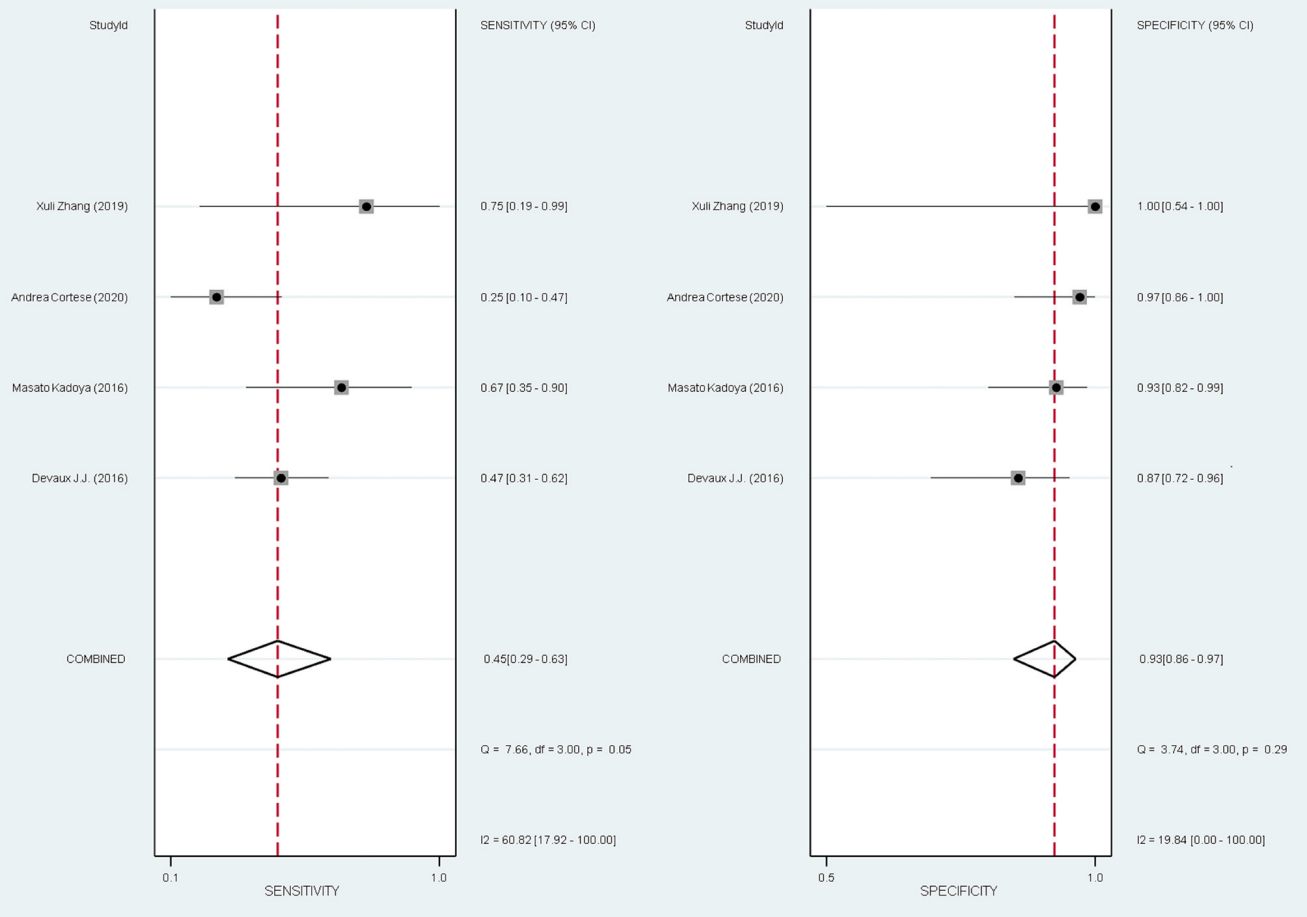
The forest plot of anti-NF155 autoantibody test in detection of a subtype of CIDP.

The overall pooled sensitivity and specificity for diagnosing the subset of CIDP with poor response to IVIg using anti-NF155 autoantibody were 0.45 (95% CI: 0.29–0.63) and 0.93 (95% CI: 0.86–0.97), respectively. These data were collated based on defining test(s) for anti-NF155 antibody as the index test and setting the EFNS/PNS criteria and poor response to IVIg as the reference standard. In this context, TP refers to the number of NF155-positive CIDP patients who responded poorly to IVIg; FP refers to the number of NF155-positive CIDP patients who responded well to IVIg; FN refers to the number of NF155-negative CIDP patients who responded poorly to IVIg; and TN refers to the number of NF155-negative participants who responded well to IVIg. Combined positive likelihood ratio (PLR) was 6.5 (95% CI: 3.3, 13.1), indicating that, when the target condition was defined as CIDP patients who responded poorly to IVIg, anti-NF155 antibody test was over 6 times more likely to correctly diagnose than misdiagnose. Combined negative likelihood ratio (NLR) was 0.59 (95% CI: 0.43, 0.80). Diagnostic odds ratio (DOR) was 11 (95% CI: 5, 26). The value of a DOR ranges from 0 to infinity, with higher values indicating better discriminatory test performance. The information was shown in Table 7, Supplementary Table 1, and Figure 6.

**Table 7:** Two-by two contingency table for diagnosis of a subtype of CIDP characterized by poor response to IVIg using anti-NF155 autoantibody.

**References**	**True positive**	**False positive**	**False negative**	**True negative**
Devaux et al. (2016)	20	5	23	33
Kadoya et al. (2016)	8	3	4	42
Zhang et al. (2019)	3	0	1	6
Cortese et al. (2020)	6	1	18	37

### Prognosis of CIDP Using Autoantibodies

**Figure 7:**
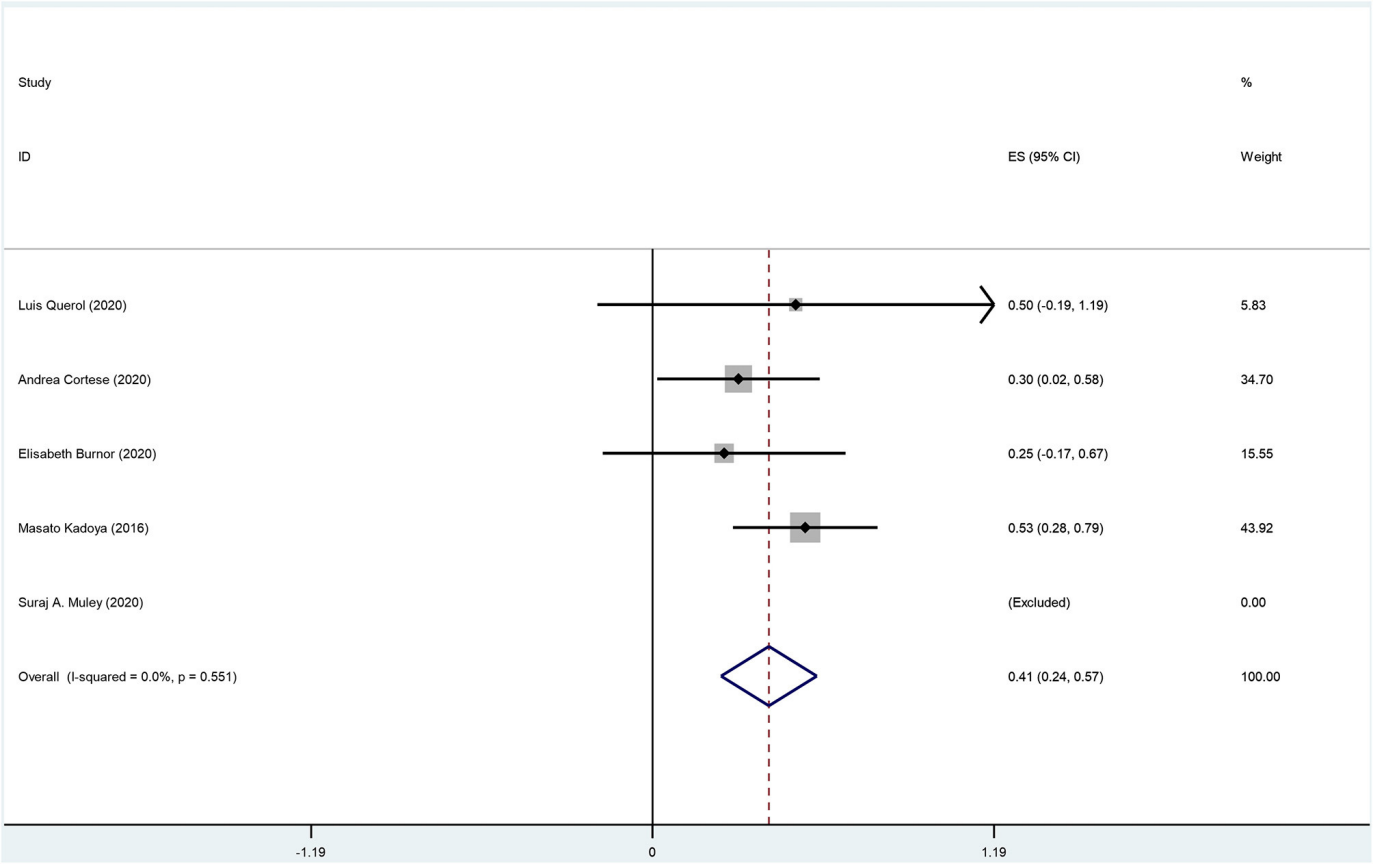
The forest plot of incidence of improvement.

**Figure 8:**
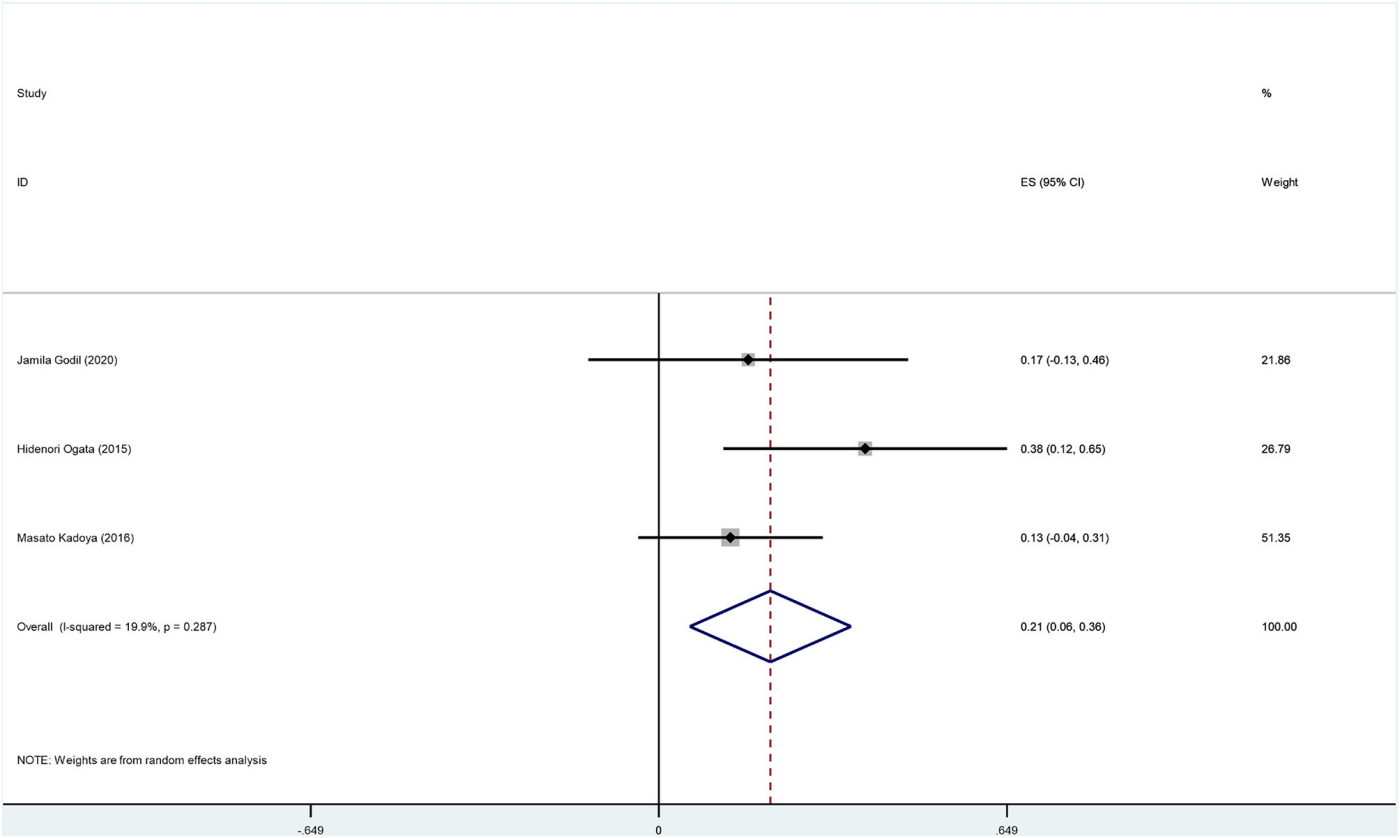
The forest plot of incidence of deterioration.

There are 8 original studies listed in Supplementary Table 2 (Ng et al., 2012; Querol et al., 2014; Ogata et al., 2015; Kadoya et al., 2016; Burnor et al., 2018; Cortese et al., 2020; Godil et al., 2020; Muley et al., 2020). Several frequently used prognosis outcomes have been listed as statistical events, such as improvement, recovery, deterioration, disability, complication, and death. The summary data are shown in Supplementary Tables 2–4 and Figures 7, 8.

The overall incidence of improvement among anti-NF155 antibody-positive CIDP patients was 0.41 (95% CI: 0.24–0.57); heterogeneity of the analysis was low; the *p*-value for the z statistic was 0.000 (<0.05), which reflects statistical significance (Supplementary Tables 2, 3 and Figure 7). It seems an improvement has been detected among nearly 41% anti-NF155 antibody seropositive CIDP patients. However, this conclusion should be interpreted with caution for two reasons: (1) the small sample size; (2) low quality of the data.

The overall incidence of deterioration among anti-NF155 antibody-positive CIDP patients was 0.20 (95% CI: 0.07–0.33); heterogeneity of the analysis was low; the *p*-value for the z statistic was 0.003 (<0.05), which reflects statistical significance (Supplementary Tables 2, 4 and Figure 8). It seems a deterioration has been detected among nearly 20% anti-NF155 antibody seropositive CIDP patients. Likewise, the result should be interpreted with caution.

## Discussion

Recent efforts have focused on the significance of the nodal and paranodal region in autoimmune responses to the nerve since the first description of paranodal autoantibodies in the serum of some CIDP patients (Ng et al., 2012). A series of observational studies, including several multicenter studies with a large sample size, were consecutively performed in academic institutions worldwide, such as KYUSHU University in Japan and the University of Wurzburg in Germany. Following preliminary searches of this topic, we aimed to systematically ingrate the newly published evidence by weighting the average results and pooling effect sizes, to resolve the discrepancies from different original studies.

We searched the database and found that Hu et al. completed a meta-analysis aimed to assess the diagnostic and therapeutic value of anti-NF155 antibody in CIDP patients (Hu et al., 2018). We conducted this new meta-analysis on the base of Hu's work. We integrated new evidence and improved Hu's approach.

Moreover, there are several limitations of Hu's meta-analysis. First, Hu et al. failed to register a study protocol on any of the prospective register platforms of systematic reviews. Hu et al. also failed to update data for more than 2 years. Hu et al. failed to maintain transparency in conducting and reporting their systematic review. This negligence may cause a higher risk of bias. Second, Hu et al. defined all included studies as cohort studies and applied the Newcastle-Ottawa scale (NOS) as a quality assessment tool. However, we reassessed some of their included studies and redefined them as cross-sectional studies, which are incompatible with the Newcastle-Ottawa rating system. Third, Hu et al. failed to rate the quality of their evidence using any approach or provide any interpretation regarding the level of evidence or recommendation strength.

We aimed to avoid the limitations mentioned above. We also aimed to apply a better designed plan. Our meta-analysis is more informative and more concise. However, incidentally, Hu's meta-analysis revealed the sensory ataxic occurrence rate and tremor occurrence rate may be higher among anti-NF155 antibody seropositive CIDP patients compared with seronegative CIDP patients. This conclusion may have clinical implication.

### Frequencies of Different Autoantibodies

The frequency of anti-NF155 autoantibody across 15 studies is 7% (95% CI: 0.05–0.10); the frequency of anti-CNTN1 autoantibody across 6 studies is 2% (95% CI: 0.01–0.03). We did not assign weights to the average percentage of other nodal/paranodal autoantibodies because of insufficient available data and a relatively low detection rate. When analyzing the frequency/positive rate of anti-NF155 antibody reported by each included studies, we found the highest three reports to be 23, 21, and 18%, collated by Yan et al. (2014), Ogata et al. (2015), and Zhang et al. (2019), respectively; the lowest three reports were 1, 2, and 4%, collated by Ng et al. (2012), Querol et al. (2014), and Kouton et al. (2020), respectively. Different antibody detection methods may cause significant variation. There are four main methods for detecting nodal/paranodal autoantibodies: ELISA, cell-based binding assay, western blot analysis, and teased nerve fiber binding assay. ELISA may show a higher sensitivity than cell-based flow cytometry in some studies, for instance (Ng et al., 2012). In addition, these various methods used for antibody detection may constitute the main part of the heterogeneity of clinical origin, as well as influence the pooled effect sizes. Furthermore, the risk of bias assessed by QUADAS-2 partially indicates the heterogeneity of methodological origin in this systematic review and meta-analysis.

### Diagnosis

The diagnosis of CIDP mainly relies on the history of symptom evaluation and characteristic feathers in nerve conduction studies. The misdiagnosis of CIDP is frequent (Allen, 2020). There is an ongoing need for diagnostic biomarkers of CIDP patients, especially for CIDP patients with anti-NF155 antibodies. In our study, anti-NF155 antibody was subjected to meta-analysis. We constructed 2 two-by-two contingency tables of true positive, true negative, false positive, and false negative rates. We want to answer the review question: what are the sensitivity and specificity of anti-NF155 antibody in diagnosing a specific subset of CIDP patients? We calculated the pooled sensitivity to be 0.45 (95% CI: 0.29–0.63) and the pooled specificity to be 0.93 (95% CI: 0.86–0.97). Consequently, the anti-NF155 antibody could be used as a specific biomarker to identify a specific subset of CIDP patients with poor response to IVIg; however, we are uncertain about the quality of evidence for this conclusion.

### Prognosis

In general, in the long term, 40% of treated CIDP patients remained dependent on treatment; severe handicap was observed in approximately one-fourth of CIDP patients (Viala et al., 2010). Despite treatment, some CIDP patients suffer from permanent neurological deficits. When comes to CIDP patients with anti-NF155 antibodies, the prognosis may be worse due to the delay of optimal treatment. There is still insufficient evidence regarding the long-term outcome of anti-NF155 antibody seropositive CIDP patients. We applied a single-rate meta-analysis to analyze the outcomes. Among 55 NF155 antibody-positive patients across 8 studies, 14 patients improved, 5 patients completely recovered, 1 patient underwent recurrence, 8 patients deteriorated, and 2 patients suffered from severe disability. Overall, the importance of follow-up data was ignored in most studies we found. Although we collected the pooled incidence of improvement (0.41, 95% CI: 0.24–0.57) and deterioration (0.20, 95% CI: 0.07–0.33) among anti-NF155 antibody seropositive CIDP patients, there were insufficient data to draw meaningful conclusions.

### Advantages

Several new observational studies have been published in the last 2 years that have not been analyzed or incorporated into the reviewed work; thus, we collated the corresponding data and obtained the combined data covering the most advanced studies of this theme. In addition, the important value of NF155 was uncovered recently and mentioned in the last updated Chinese clinical guideline, which we considered to be of significant value for helping clinical decisions. To date, few systematic works have been applied to this theme, at least not covering both diagnostic and prognostic perspectives, as mentioned above constituted the original intention of the present work. Furthermore, the innovation of our work focused on the methodology for analyzing diagnostic data. The single-arm meta-analyses were used to analyze the data of levels and frequency of antibodies and the data of the prognosis section.

All the authors were trained for evidence-based methodology before conducting this review. We registered the protocol on the most common academic register and strictly followed the PRISMA-P guidelines. The process was transparent.

Our work screened 10,638 studies and incorporated a detailed analysis of 20 studies to rate the frequencies of nodal/paranodal autoantibodies, the diagnostic accuracy of autoantibody testing, and the prognostic value for CIDP. These conclusions relate to both the methodological conduct of biomarker studies for CIDP and the consideration of new immunotherapies such as rituximab and cyclophosphamide for CIDP patients. We preliminarily integrate evidence with the utilization of testing autoantibodies against the paranode, for further studies and decisions, such as cost benefit analysis.

### Limitations

Despite these advantages, there are several limitations to this systematic review that are relevant when using this review as a reference for evidence-informed health policymaking.

First, we did not use the grading of recommendations, assessment, development, and evaluation (GRADE) approach for rating the certainty and quality of evidence for each outcome. To our knowledge, the methodology for systematically reviewing observational studies is still developing. The observational studies we included in this systematic review are typically considered low-quality evidence. Thus, we believe that further research is very likely to have a significant impact on our present conclusions.

In addition, regarding the diagnostic section that assessed the diagnostic value of autoantibodies, we strictly followed the protocol CRD42020203385. There was very little difference between that protocol and the diagnostic section of this systematic review. However, for the prognostic section assessing the prognostic value of autoantibodies, we could not assess the data of all the studies that we identified and preferred to include in our review. The lack of available data forced us to abandon some of the preferred intended outcomes in protocol CRD42020190789. Several differences cannot be ignored between protocol CRD42020190789 and the prognostic section of this systematic review.

## Conclusions

CIDP is a rare immune-mediated heterogeneous disease characterized by demyelination of the peripheral nervous system. Diagnosis of CIDP relies on clinical and neurophysiologic criteria and lacks useful diagnostic biomarkers. Autoantibodies against paranodal proteins have been reported in some patients diagnosed with CIDP. These patients have atypical clinical phenotypes and impaired response to conventional treatments. These autoantibodies' targets include NF155, CNTN1, CASPR1, and Ranvier's nodal proteins NF140 and NF186. We conducted a meta-analysis to summarize evidence on the diagnostic and prognostic value of these autoantibodies for a subset of CIDP, as well as to uncover the frequencies of these autoantibodies.

When it comes to diagnosing a specific subset of CIDP characterized by poor response to IVIg, we found a moderate sensitivity and a high specificity. The pooled frequency of anti-NF155 antibodies in our meta-analysis was 7%; the frequency of CNTN1 was 2%. Considering the bias between the pooled data and validity, we suggest a cautious interpretation of the presented results. The prognostic value of anti-NF155 autoantibody was uncertain due to the sample size limitation and single-arm meta-analysis methodology.

We synthesized the evidence to promote good decision making in the clinical care of CIDP; we also aimed to assess the value of neurofascin antibodies and other autoantibodies in diagnosing CIDP and in guiding treatment. We suggest further large cohort studies exploring the association between refractory patients with CIDP and autoantibodies against the nodal or paranodal proteins.

## Data Availability Statement

The original contributions presented in the study are included in the article/Supplementary Material, further inquiries can be directed to the corresponding author/s.

## Author Contributions

XT conceived, designed, and supervised the systematic review. XG and LT contributed significantly to the data analysis and manuscript preparation. XG drafted the manuscript. QH contributed to discussions and manuscript revision. All authors approved the final version and agreed to be accountable for all aspects of the work.

## Conflict of Interest

The authors declare that the research was conducted in the absence of any commercial or financial relationships that could be construed as a potential conflict of interest.
