# Supplementary material for: A Systematic Review and Meta-Analysis of Autoantibodies for Diagnosis and Prognosis in Patients With Chronic Inflammatory Demyelinating Polyradiculoneuropathy
Source: Front Neurosci. 2021 May 24;15:637336. doi: 10.3389/fnins.2021.637336 (PMC8180587; doi:10.3389/fnins.2021.637336)
Supplement: Supplementary file 1 [file Data_Sheet_1.PDF]

Supplementary Table 1. Illustration of Figure 6

Parameter	Estimate	95% CI
Sensitivity	0.45	[0.29, 0.63]
Specificity	0.93	[0.86, 0.97]
Positive Likelihood Ratio	6.5	[3.3, 13.1]
Negative Likelihood Ratio	0.59	[0.43, 0.80]
Diagnostic Odds Ratio	11	[5, 26]

Note How do we plot Figure 6? We use STATA 16.0 to calculate the combined sensitivity, specificity, positive likelihood ratio (PLR), negative likelihood ratio (NLR) and diagnostic odds ratio (DOR).

Supplementary Table 2. Summary data for prognostic section of this systematic review and meta-analysis

Author (Year)	Total	Improvement	Recovery	Recurrence	Deterioration	Disability	Complication	Death	Unknown
Judy King Man Ng (2012)	**4**		**1**						**3**
Luis Querol (2014)	**2**	**1**		**1**					
Hidenori Ogata (2015)	**13**				**5**				**8**
Masato Kadoya (2016)	**15**	**8**			**2**				**5**
Elisabeth Burnor (2018)	**4**	**1**				**2**			**1**
Andrea Cortese (2020)	**10**	**3**							**7**
Suraj A. Muley (2020)	**1**	**1**							
Jamila Godil (2020)	**6**		**4**		**1**				**1**

Note Total=total number of NF155 positive patients in the original study; several frequently used outcomes of prognosis have been listed as statistical events; some data directly referred to the data written in the original papers, however, other data were inferred from the context.

Supplementary Table 3. The illustration of Figure 7

Study	ES (effect size)	[95% Conf. Interval]		% Weight
Masato Kadoya (2016)	0.533	0.281	0.786	43.92
Elisabeth Burnor (2018)	0.250	-0.174	0.674	15.55
Luis Querol (2020)	0.500	-0.193	1.193	5.83
Andrea Cortese (2020)	0.300	0.016	0.584	34.70
Suraj A. Muley (2020)	Excluded			
D+L pooled ES	0.406	0.239	0.574	100.00

Heterogeneity chi-squared=2.10 (df= 3); p=0.551; I-squared (variation in ES attributable to heterogeneity) =0.0%; Estimate of between-study variance Tau-squared =0.0000; Test of ES=0: z=4.76; p=0.000

Supplementary Table 4. The illustration of Figure 8

Study	ES (effect size)	[95% Conf. Interval]		% Weight
Hidenori Ogata (2015)	0.385	0.120	0.649	24.10
Masato Kadoya (2016)	0.133	-0.039	0.305	56.95
Jamila Godil (2020)	0.167	-0.132	0.465	18.95
D+L pooled ES	0.200	0.070	0.330	100.00

Heterogeneity chi-squared=2.50 (df=2); p=0.287; I-squared (variation in ES attributable to heterogeneity) =19.9%; Estimate of between-study variance Tau-squared=0.003; Test of ES=0: z=3.02; p = 0.003
