# Supplementary material for: A Systematic Review and Meta-Analysis of Autoantibodies for Diagnosis and Prognosis in Patients With Chronic Inflammatory Demyelinating Polyradiculoneuropathy
Source: Front Neurosci. 2021 May 24;15:637336. doi: 10.3389/fnins.2021.637336 (PMC8180587; doi:10.3389/fnins.2021.637336)
Supplement: Supplementary file 2 [file Data_Sheet_2.PDF]

Search Strategy for Identification of Studies

Review Title: Explore the correlation between IgG4 antibodies against (para)nodal proteins and prognosis of patients with chronic inflammatory demyelinating polyradiculoneuropathy via meta-analysis.

Records identified through database searching (n = 2849)
CENTRAL = 52
PubMed = 1169
EMBASE = 1460
Web of Science = 168

CENTRAL Search Strategy
Records after duplicates removed (n = 52)

#1	MeSH descriptor: [Immunoglobulin G4-Related Disease] explode all trees
#2	MeSH descriptor: [Cell Adhesion Molecules, Neuronal] explode all trees
#3	MeSH descriptor: [Antibodies] explode all trees
#4	(IgG4):ti,ab,kw (Word variations have been searched)
#5	(neurofascin):ti,ab,kw (Word variations have been searched)
#6	(neurofascin*):ti,ab,kw (Word variations have been searched)
#7	("neurofascin 155"):ti,ab,kw (Word variations have been searched)
#8	(NF*):ti,ab,kw (Word variations have been searched)
#9	("NF 155 protein"):ti,ab,kw (Word variations have been searched)
#10	#1 or #2 or #3 or #4 or #5 or #6 or #7 or #8 or #9
#11	MeSH descriptor: [Polyradiculoneuropathy, Chronic Inflammatory Demyelinating] explode all trees
#12	("Polyradiculopathy, Chronic Inflammatory"):ti,ab,kw (Word variations have been searched)
#13	(CIDP):ti,ab,kw OR ("chronic inflammatory polyradiculoneuropathy"):ti,ab,kw OR ("chronic inflammatory demyelinating polyradiculoneuropathy"):ti,ab,kw OR (Polyradiculoneuropathy Chronic Inflammatory):ti,ab,kw OR (Chronic Inflammatory Polyradiculopathies):ti,ab,kw (Word variations have been searched)
#14	(Chronic Inflammatory Polyradiculopathy):ti,ab,kw OR (Polyneuropathy Inflammatory Demyelinating Chronic):ti,ab,kw OR (CADP):ti,ab,kw OR (chronic acquired demyelinating polyneuropathy):ti,ab,kw OR (distal acquired demyelinating symmetric neuropathy):ti,ab,kw (Word variations have been searched)
#15	(Polyradiculopathy Chronic Inflammatory):ti,ab,kw (Word variations have been searched)
#16	(DADS):ti,ab,kw OR (multifocal acquired demyelinating sensory and motor neuropathy):ti,ab,kw OR (MADSAM):ti,ab,kw OR (Lewis Sumner syndrome):ti,ab,kw OR (chronic guillain barre syndrom):ti,ab,kw (Word variations have been searched)
#17	(auto immune polyneuropathy):ti,ab,kw OR (chronic guillain barre syndrome):ti,ab,kw (Word variations have been searched)
#18	(Polyradiculopathies Chronic Inflammatory):ti,ab,kw OR (Inflammatory

	Polyradiculopathies Chronic):ti,ab,kw OR (Chronic Inflammatory Polyradiculoneuropathies):ti,ab,kw OR (Polyradiculoneuropathies Chronic Inflammatory):ti,ab,kw OR (Inflammatory Polyradiculopathy Chronic):ti,ab,kw (Word variations have been searched)
#19	#11 or #12 or #13 or #14 or #15 or #16 or #17 or #18
#20	#10 and #19

PubMed Search Strategy

Records (n = 1169)

#1	Chronic Inflammatory Demyelinating Polyradiculoneuropathy[MeSH Terms]
#2	Polyradiculoneuropathy, Chronic Inflammatory Demyelinating[MeSH Terms]
#3	CIDP[Title/Abstract]
#4	Polyneuropathy, Inflammatory Demyelinating Chronic[Title/Abstract]
#5	Inflammatory Polyradiculopathy Chronic[Title/Abstract]
#6	Chronic Inflammatory Polyradiculopathies[Title/Abstract]
#7	Chronic Inflammatory Polyradiculopathy[Title/Abstract]
#8	Inflammatory Polyradiculopathies Chronic[Title/Abstract]
#9	Polyradiculopathies Chronic Inflammatory[Title/Abstract]
#10	Polyradiculopathy Chronic Inflammatory[Title/Abstract])) OR (Polyradiculoneuropathy Chronic Inflammatory[Title/Abstract]
#11	Chronic Inflammatory Polyradiculoneuropathy[Title/Abstract]
#12	Chronic Inflammatory Polyradiculoneuropathies[Title/Abstract]
#13	Polyradiculoneuropathies Chronic inflammatory[Title/Abstract]
#14	Chronic Inflammatory Demyelinating Polyradiculoneuropathy[Title/Abstract]
#15	CADP[Title/Abstract]
#16	chronic acquired demyelinating polyneuropathy[Title/Abstract]
#17	distal acquired demyelinating symmetric neuropathy[Title/Abstract]
#18	DADS[Title/Abstract]
#19	multifocal acquired demyelinating sensory[Title/Abstract]
#20	motor neuropathy[Title/Abstract]
#21	MADSAM[Title/Abstract]
#22	Lewis Sumner syndrome[Title/Abstract]
#23	chronic guillain barre syndrome[Title/Abstract]
#24	auto?immune polyneuropathy[Title/Abstract]
#25	#1 or #2 or #3 or #4 or #5 or #6 or #7 or #8 or #9 or #10 or #11 or #12 or #13 or #14 or #15 or #16 or #17 or #18 or #19 or #20 or #21 or #22 or #23 or #24
#26	antibodies[MeSH Terms]
#27	neurofascin protein human[MeSH Terms]
#28	neurofascin155 protein human[MeSH Terms]
#29	NF 155 protein human[MeSH Terms]
#30	neurofascin[Title/Abstract]
#31	IgG4 antoantibodies[Title/Abstract]

#32	IgG4[Title/Abstract]
#33	NF155[Title/Abstract]
#34	immunoglobulin G4[Title/Abstract]
#35	immunoglobulin G4 antibodies[Title/Abstract]
#36	nodal proteins[Title/Abstract]
#37	paranodal proteins[Title/Abstract]
#38	antibodies[Title/Abstract]
#39	#26 or #27 or #28 or #29 or #30 or #31 or #32 or #33 or #34 or #35 or #36 or #37 or #38
#40	#25 and #39

EMBASE Search Strategy
Records (n = 1460)

#1	cidp:ab,ti OR 'polyneuropathy,inflammatory demyelinating,chronic':ab,ti OR 'inflammatory polyradiculopathy,chronic':ab,ti OR 'chronic inflammatory polyradiculopathies':ab,ti OR 'chronic inflammatory polyradiculopathy':ab,ti OR 'inflammatory polyradiculopathies,chronic':ab,ti OR 'polyradiculopathies,chronic inflammatory':ab,ti OR 'polyradiculopathy,chronic inflammatory':ab,ti OR 'polyradiculoneuropathy,chronic inflammatory':ab,ti OR 'chronic inflammatory polyradiculoneuropathy':ab,ti OR 'chronic inflammatory polyradiculoneuropathies':ab,ti OR 'polyradiculoneuropathies,chronic inflammatory':ab,ti OR 'chronic inflammatory demyelinating polyradiculoneuropathy':ab,ti
#2	cadp:ab,ti OR 'chronic acquired demyelinating polyneuropathy':ab,ti OR 'distal acquired demyelinating symmetric neuropathy':ab,ti OR dads:ab,ti OR ('multifocal acquired demyelinating sensory':ab,ti AND 'motor neuropathy':ab,ti) OR madsam:ab,ti OR 'lewis sumner syndrome':ab,ti OR 'chronic guillain barre syndrome':ab,ti OR 'auto immune polyneuropathy':ab,ti
#3	#1 or #2
#4	'chronic inflammatory demyelinating polyradiculopathy'/exp OR 'chronic inflammatory demyelinating neuropathy'/exp OR 'chronic inflammatory demyelinating polyneuropathy'/exp OR 'chronic inflammatory demyelinating polyradiculoneuropathy'/exp
#5	#3 or #4
#6	'antibody'/exp OR 'neurofascin'/exp OR 'neurofascin 155'/exp OR 'neurofascin 186'/exp OR 'neurofascin 155 antibody'/exp OR 'neurofascin antibody'/exp
#7	neurofascin:ab,ti OR 'igg4 autoantibodies' OR nf155:ab,ti OR 'immunoglobulin g4':ab,ti OR 'immunoglobulin g4 antibodies':ab,ti OR'nodal proteins':ab,ti OR 'paranodal proteins':ab,ti OR contactin:ab,ti OR caspr:ab,ti OR cntn:ab,ti OR 'contactin associated protein':ab,ti OR igg4:ab,ti
#8	#6 or #7
#9	#5 and #8

Web of Science Search Strategy
Records (n = 168)

#1	TS:(CIDP) OR TS:(Polyneuropathy Inflammatory Demyelinating Chronic) OR TS:(inflammatory Polyradiculopathy Chronic) OR TS:(Chronic Inflammatory Polyradiculopathies) OR TS:(Chronic Inflammatory Polyradiculopathy) OR TS:(Inflammatory Polyradiculopathies Chronic) OR TS:(Polyradiculopathies Chronic Inflammatory) OR TS:(Polyradiculopathy Chronic Inflammatory) OR TS:(Polyradiculoneuropathy Chronic Inflammatory) OR TS:(Chronic Inflammatory Polyradiculoneuropathy) OR TS:(Chronic Inflammatory Polyradiculoneuropathies) OR TS:(Polyradiculoneuropathies Chronic Inflammatory) OR TS:(Chronic Inflammatory Demyelinating Polyradiculoneuropathy)
#2	TS:(CADP) OR TS:(chronic acquired demyelinating polyneuropathy) OR TS:(distal acquired demyelinating symmetric neuropathy) OR TS:(DADS) OR TS:(multifocal acquired demyelinating sensory and motor neuropathy) OR TS:(MADSAM) OR TS:(Lewis Sumner syndrome) OR TS:(chronic guillain barre syndrome) OR TS:(auto immune polyneuropathy)
#3	#2 or #1
#4	TS:(neurofascin) OR TS:(IgG4 autoantibodies) OR TS:(NF155) OR TS:(immunoglobulin G4) OR TS: (immunoglobulin G4 antibodies) OR TS:(nodal proteins) OR TS:(paranodal proteins) OR TS:(neurofascin protein) OR TS:(neurofascin 155 protein) OR TS:(NF155 protein)
#5	#4 and #3
